# Supplementary figures and images for: Hypoxia‐mediated regulation of DDX5 through decreased chromatin accessibility and post‐translational targeting restricts R‐loop accumulation
Source: Mol Oncol. 2023 Apr 22;17(7):1173–91. doi: 10.1002/1878-0261.13431 (PMC10323886; doi:10.1002/1878-0261.13431)

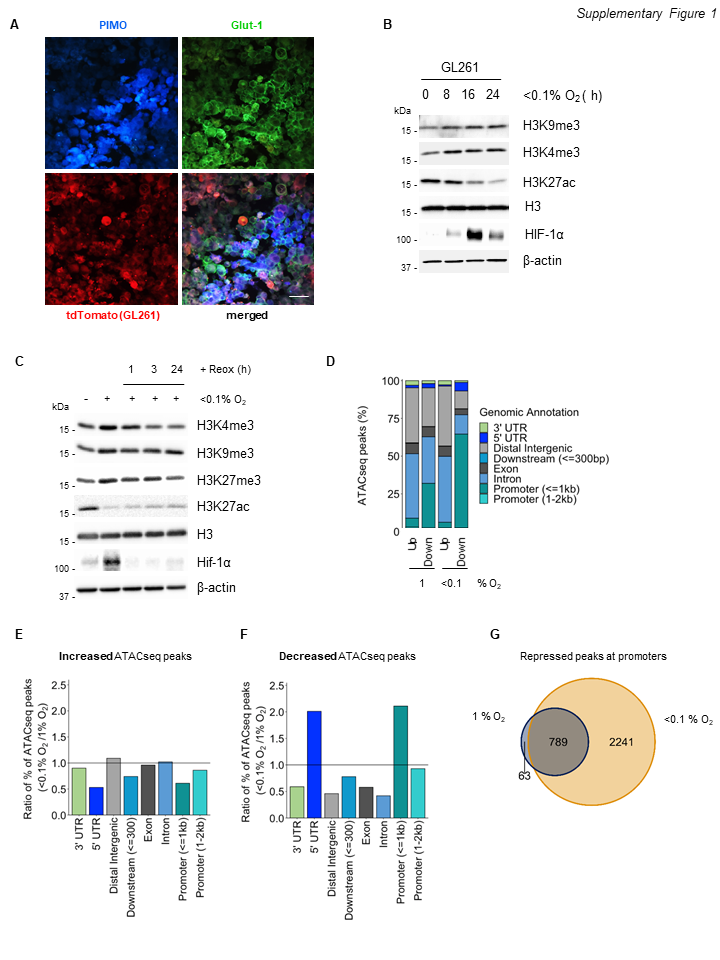

Supplement: Supplementary file 1 — Fig. S1. Oxygen‐dependent changes in the chromatin accessibility. [file MOL2-17-1173-s011.tif]

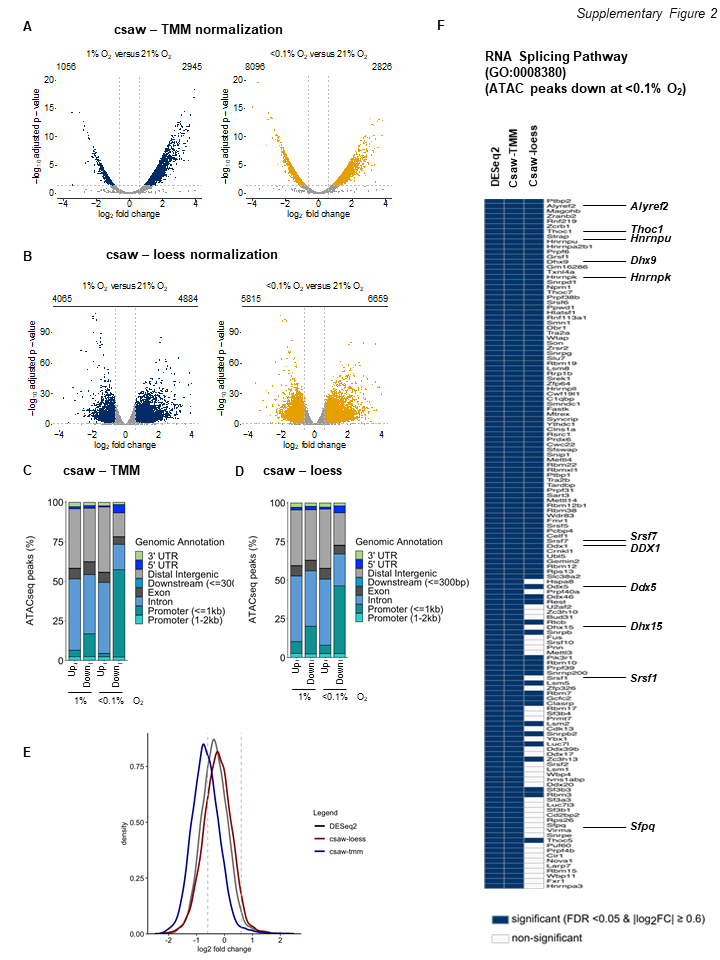

Supplement: Supplementary file 2 — Fig. S2. ATACseq data analysis using alternative normalization methods. [file MOL2-17-1173-s009.TIF]

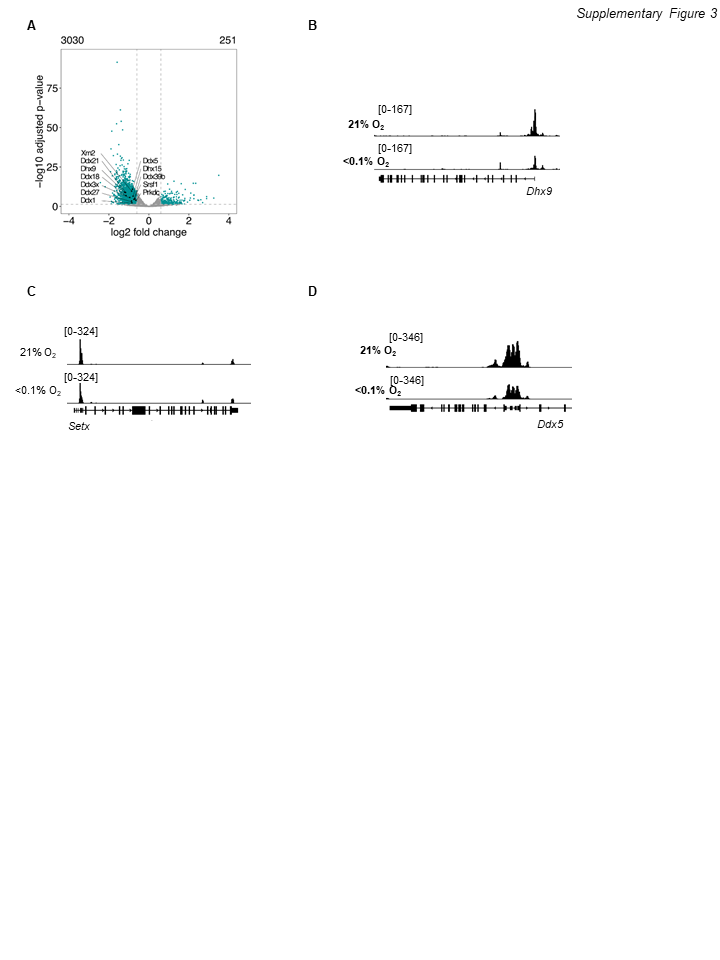

Supplement: Supplementary file 3 — Fig. S3. Hypoxia‐dependent chromatin accessibility changes at R‐loop interacting factors. [file MOL2-17-1173-s008.TIF]

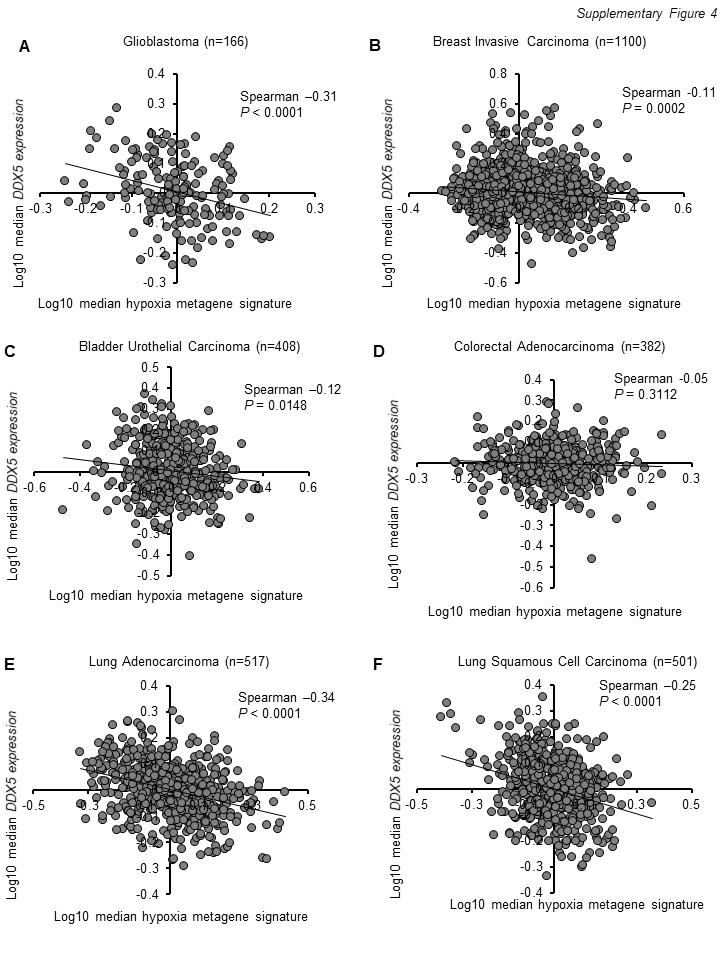

Supplement: Supplementary file 4 — Fig. S4. Correlation between DDX5 expression and hypoxia metagene in TCGA cancer patient samples. [file MOL2-17-1173-s012.TIF]

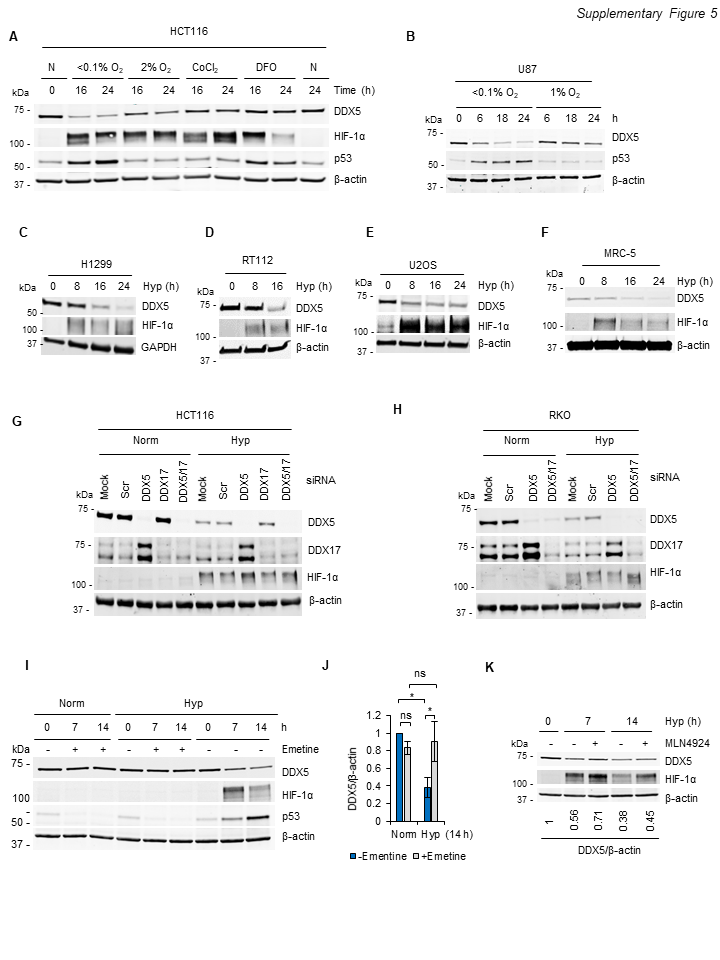

Supplement: Supplementary file 5 — Fig. S5. Hypoxia‐dependent DDX5 repression in multiple cell lines. [file MOL2-17-1173-s003.tif]

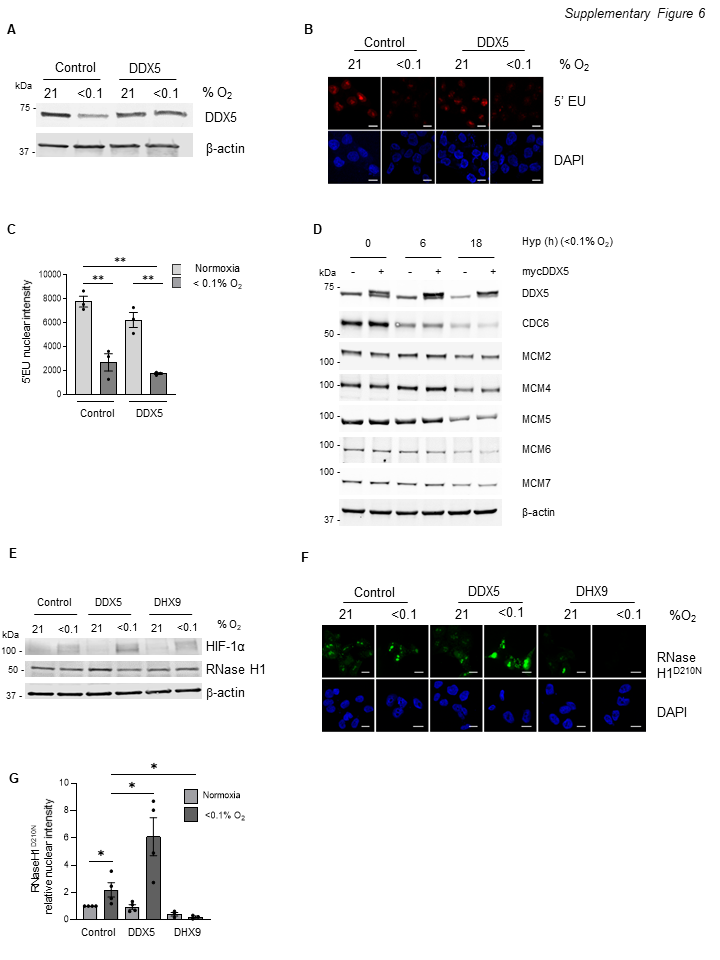

Supplement: Supplementary file 6 — Fig. S6. Effects of rescue of DDX5 expression in hypoxia on transcription and R‐loops in HCT116 cells. [file MOL2-17-1173-s005.tif]
